# CoMnO_x_ Nanoflower-Based Smartphone Sensing Platform and Virtual Reality Display for Colorimetric Detection of Ziram and Cu^2+^

**DOI:** 10.3390/bios14040178

**Published:** 2024-04-06

**Authors:** Chang Song, Fangfang Wang, Xin Zhang, Yuanxia Ma, Yangyu Wu, Mingxia He, Xiangheng Niu, Mengmeng Sun

**Affiliations:** 1 School of Arts and Media, Sichuan Agricultural University, Chengdu 611130, China; 2 College of Science, Sichuan Agricultural University, Ya’an 625014, China; 3 School of Public Health, Hengyang Medical School, University of South China, Hengyang 421001, China

**Keywords:** cobalt manganese oxide, oxidase-like activity, ziram, Cu^2+^, intelligent detection

## Abstract

Transition metal doping is an ideal strategy to construct multifunctional and efficient nanozymes for biosensing. In this work, a metal-doped CoMnO_x_ nanozyme was designed and synthesized by hydrothermal reaction and high-temperature calcination. Based on its oxidase activity, an “on-off-on” smartphone sensing platform was established to detect ziram and Cu^2+^. The obtained flower-shaped CoMnO_x_ could exhibit oxidase-, catalase-, and laccase-like activities. The oxidase activity mechanism of CoMnO_x_ was deeply explored. O_2_ molecules adsorbed on the surface of CoMnO_x_ were activated to produce a large amount of O_2_^·-^, and then, O_2_^·-^ could extract acidic hydrogen from TMB to produce blue oxTMB. Meanwhile, TMB was oxidized directly to the blue product oxTMB via the high redox ability of Co species. According to the excellent oxidase-like activity of CoMnO_x_, a versatile colorimetric detection platform for ziram and Cu^2+^ was successfully constructed. The linear detection ranges for ziram and Cu^2+^ were 5~280 μM and 80~360 μM, and the detection limits were 1.475 μM and 3.906 μM, respectively. In addition, a portable smartphone platform for ziram and Cu^2+^ sensing was established for instant analysis, showing great application promise in the detection of real samples including environmental soil and water.

## 1. Introduction

Zinc dimethyl dithiocarbamate (ziram) is a widely used organosulfur fungicide that can inhibit and prevent diseases caused by a variety of fungi. However, due to the overuse and abuse of ziram, it often leads to pesticide residues in food and water, causing serious effects on human health. With the increase of living standards, pesticide residue in food has become a hot issue in society, and research on pesticide residue detection has become popular. Currently, common methods for the determination of pesticide residues include high-performance liquid chromatography (HPLC) [1], spectrophotometry [2], capillary electrophoresis [3], and voltammetry [4]. However, all of the above methods are not suitable for in-field analysis, severely constraining the widespread use of the above techniques for detecting pesticide residues on site and in a timely manner. Simple, rapid, and sensitive modes for detecting pesticide residues need to be developed.

Nanozymes are nanomaterials that can catalyze enzyme substrates under mild or extreme conditions and convert the substrates into products following enzyme kinetics. Since the first study of Fe_3_O_4_ nanomaterials with horseradish peroxidase (HRP) catalytic activity was reported in 2007 [5], great effort has been made to develop nanomaterials with similar catalytic activity [6,7,8,9]. Nanozymes present a lower cost, higher stability, higher adaptation to extreme conditions, and higher recovery efficiency than natural enzymes. In recent years, metals [10,11], metal composites [12,13,14], and carbon-based materials [15] have all been discovered and designed as nanomaterials with enzyme-like catalytic effects. Nanozymes have been extensively studied in biosensors, environmental protection, disease diagnosis, and antimicrobial agents [16,17,18]. Among all the various nanozymes, redox nanozymes are the most studied. According to different catalytic types, redox nanozymes often present many kinds of simulated enzyme activities, such as peroxidase- (POD), oxidase- (OXD), catalase- (CAT), and superoxide dismutase-like (SOD) activities [19,20,21,22]. Most research in the field currently focuses on peroxidase-like activity, whereas oxidase-like activity receives much less attention [23,24,25,26]. In the process of catalysis by peroxidase mimics, external H_2_O_2_ is required to act as an electron acceptor. For oxidase catalysis, dissolved O_2_ can be used as a substrate, so the catalytic operation is more direct and simpler. As a result, oxidase-like nanozymes act as a compelling option for creating sensors with a straight-forward operation, excellent compatibility, and high reliability.

Manganese oxides (MnO_x_) have been widely used to detect various small biomolecules based on their oxidase activity, including hydroquinone [27], organophosphorus [28], and ascorbic acid [29]. Manganese oxides exist in more than 30 different natural crystal forms, and these minerals are important constituents of sediments and soils, participating in various natural chemical reactions [30]. However, manganese oxides have poor catalytic performance, and they are difficult to separate after the reaction. To better mimic natural enzymes, the catalytic activity of nanozymes can be designed by tuning size, morphology, composition, surface functional groups, and exposed faces. Heteroatom doping is a good strategy to enhance the activity of nanozymes. Transition metal-doped nanozymes can exhibit higher catalytic activity than pristine materials [31]. These materials show superior catalytic activity to monometallic materials due to electronic structural effects and synergistic effects.

In this study, CoMnO_x_ with multiple enzyme-like catalytic properties was designed to construct an “on-off-on” sensing platform for ziram and Cu^2+^. CoMnO_x_ was prepared by hydrothermal and calcination methods. The oxidase-, catalase-, and laccase-like activities of the obtained CoMnO_x_ were evaluated, and the reaction kinetics and mechanisms were studied in detail. The substrate TMB could be oxidized to oxTMB by a large amount of O_2_^·-^ generated from oxidase-like catalysis. Meanwhile, it was also oxidized directly to the blue product oxTMB via the high redox ability of Co species. Based on the oxidase activity of CoMnO_x_, a multifunctional colorimetric sensing platform for ziram and Cu^2+^ was established, which provided wide detection ranges and low detection limits. The combination of the sensing method and a portable smartphone was further made to achieve real-time detection, and the application potential in monitoring ziram and Cu^2+^ in environmental soil and water was also assessed.

## 2. Materials and Methods

### 2.1. Preparation of CoMnO_x_

Co(NO_3_)_2_‧6H_2_O (0.4366 g) and KMnO_4_ (0.2371 g) were gradually put into 21 mL of deionized water with strong stirring. After stirring for 10 min, the mixture was autoclaved in a 30 mL autoclave. After autoclaving, the mixture was transferred into an oven and kept for 6 h at 150 °C. After the heated treatment, the formed product was precipitated to obtain solid powders. The collected solid powders were washed several times by deionized water and ethanol, respectively. Lastly, the powders were treated in a vacuum oven for 12 h. After drying, a certain amount of the solid powders was put into a tube furnace and annealed at 450 °C for 30 min under an argon atmosphere. After calcination, the proposed CoMnO_x_ was obtained.

### 2.2. Enzyme-Like Activities of CoMnO_x_

Firstly, the OXD-like activity of CoMnO_x_ was investigated with a conventional method. 3,3’,5,5’-Tetramethylbenzidine (TMB) was used as a chromogenic substrate to evaluate the OXD-like activity. During tests, 210 μL of TMB (5 mM) and 150 μL of CoMnO_x_ (1 mg·mL^−1^) were mixed with 0.2 M of HAc-NaAc buffer (the total volume was 3 mL, pH 4.5). Afterwards, the mixture was kept in a water bath at 40 °C for 20 min. After that, the mixture solution was used to investigate the absorbance at 652 nm by an ultraviolet-visible (UV-Vis) spectrophotometer.

To verify the CAT-like activity of CoMnO_x_, the mixture solution was prepared by mixing Tris-HCl buffer (pH 8.0), 100 μL of CoMnO_x_ (1 mg·mL^−1^), and 200 μL of H_2_O_2_ (20 mM). After the mixture was kept in a water bath at 25 °C for 10 min, the absorbance at 240 nm was obtained by UV-Vis as time went by.

To characterize the laccase-like activity of CoMnO_x_, the mixture solution was prepared by mixing 50 mM of MES buffer (pH 7.0), 100 μL of 2,4-DCP (1.0 mg·mL^−1^), and 4-AP. The volume of the mixture solution was 1.5 mL. Afterwards, a CoMnO_x_ suspension was introduced and the mixture was incubated for 90 min at 37 °C. Finally, the solution absorbance was obtained by UV-Vis.

### 2.3. Steady-State Kinetic Study

After condition optimization experiments, the catalytic property and kinetic parameters of CoMnO_x_ with oxidase-like activity were investigated. Firstly, a series of mixtures were prepared by mixing 0.2 M of HAc-NaAc buffer (pH 4.5) and 150 μL of CoMnO_x_ (1 mg·mL^−1^) with different concentrations of TMB. Then, a series of mixtures were kept in a water bath at 40 °C for 20 min. The oxidase-like activity of these mixtures was obtained at 652 nm under optimized conditions by UV-Vis. Based on the Michaelis–Menten equation, *K*_m_ and *V*_max_ were obtained.

1/V = (*K*_m_/*V*_max_)(1/[S]) + 1/*V*_max_

where *K*_m_ is the Michaelis constant, [S] is the substrate concentration, and *V*_max_ is the maximum reaction velocity.

### 2.4. Oxidase-Like Reaction Mechanism

Different radical scavengers were employed to investigate reactive oxygen species (ROS) formed during the oxidase-like reaction. The mixture solution was obtained by mixing 100 µL of different concentrations of scavengers, 210 μL of TMB, 150 μL of CoMnO_x_, and HAc-NaAc buffer. Hydroxyl radical (·OH), singlet oxygen (^1^O_2_), and superoxide anion (O_2_^−^) were measured by the radical scavengers of isopropyl alcohol (IPA), NaN_3_, and 1,4-benzoquinone (PBQ), respectively. Oxygen vacancies were tested by adding EDTA. After incubating, the oxidase-like activity of the mixture was tested at 652 nm.

### 2.5. Colorimetric Detection of Ziram and Cu^2+^

For the colorimetric detection of ziram, 210 μL of TMB and 150 μL of CoMnO_x_ were mixed with HAc-NaAc buffer. Then, the effects of various concentrations of ziram on the oxidase-like activity of the mixture were investigated. After incubating, the intensity of absorbance at 652 nm was tested by UV-Vis.

For the colorimetric detection of Cu^2+^, 480 μL of ziram (4 mM) and various Cu^2+^ concentrations were mixed with the HAc-NaAc buffer system containing 210 μL of TMB and 150 μL of CoMnO_x_. After incubating at 40 °C, the effects of various concentrations of Cu^2+^ on the absorbance were investigated, and the intensity of absorbance at 652 nm was tested by UV-Vis.

### 2.6. Visual Smartphone Detection Platform

Based on the colorimetric detection of ziram and Cu^2+^, a portable smartphone platform was established for instant analysis. Firstly, a large number of photos were collected, and then the red-green-blue (RGB) and hue-saturation-lightness (HSL) of the photos were extracted and trained by deep learning. As for the intelligent detection of ziram and Cu^2+^, the photos of colorimetric results were uploaded to a smartphone, and the values of RGB and HSL could be recognized automatically by an artificial intelligence program. The values or their combinations of RGB/HSL and the concentrations of targets were used to establish standard curves by the smartphone. At the same time, the results of the linear equation and correlation coefficient were formed automatically.

### 2.7. Real Sample Analysis

To investigate the practicability of the developed sensing platform based on the oxidase-like activity of CoMnO_x_, a standard addition method was employed. The supernatants of river water and soil were obtained after centrifugation treatment. Before testing, the obtained liquids were diluted 100 times. Certain concentrations of Cu^2+^ and ziram were added into the liquids. The color photos were taken and uploaded to the smartphone platform. The values of RGB and HSL could be recognized automatically by the artificial intelligence program, and the corresponding concentrations of Cu^2+^ and ziram were output automatically.

## 3. Results

### 3.1. Synthesis and Characterization of CoMnO_x_ Nanoflowers

**Figure 1:**
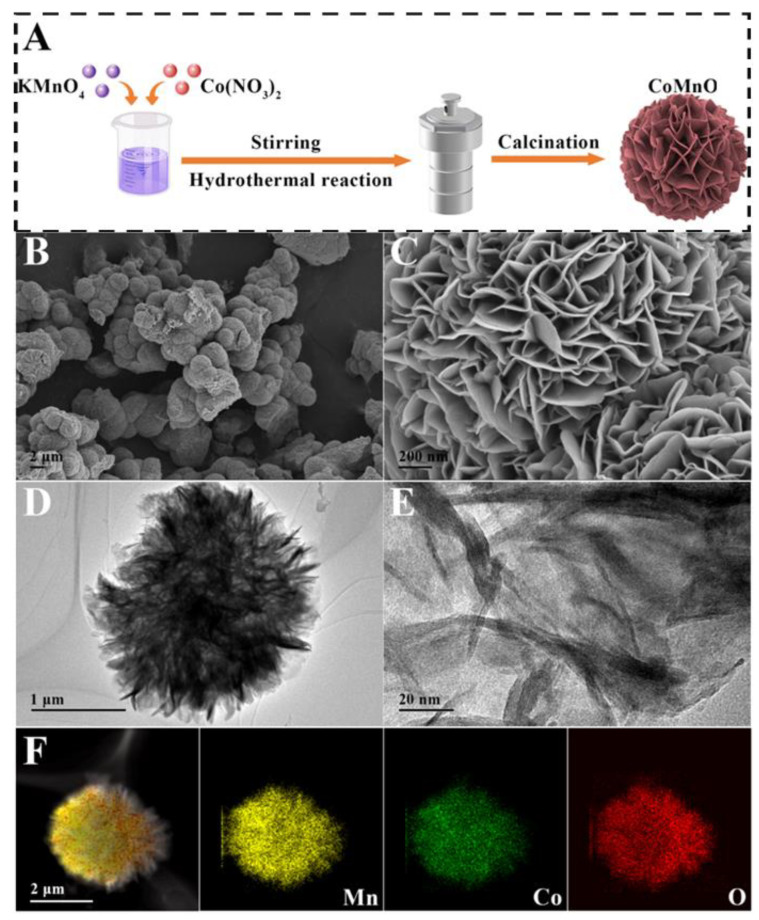
Synthesis and characterization of CoMnO_x_ nanoflowers. (**A**) Synthesis diagram; (**B**,**C**) scanning electron microscopy (SEM) of CoMnO_x_ nanoflowers; (**D**,**E**) transmission electron microscopy (TEM) of CoMnO_x_ nanoflowers; (**F**) elemental mapping pattern of CoMnO_x_ nanoflowers.

The synthesis diagram of CoMnO_x_ is presented in Figure 1A. The samples of CoMnO_x_ were prepared by hydrothermal synthesis and high-temperature calcination. Figure 1B–E show that the obtained CoMnO_x_ has a flower-like shape with rich petal wrinkles. The possible mechanism for CoMnO_x_ forming such a flower-like structure is MnO_x_ crystal nucleus growth during the Ostwald ripening process [32]. The distribution of elements is displayed in Figure 1F. The results reveal that Co, Mn, and O elements disperse homogeneously. The X-ray diffraction (XRD) pattern of CoMnO_x_ presents diffraction peaks of (111), (220), and (422) (Figure S1), which are attributed to MnO_x_ (PDF# 21-0547). As shown in Figure S1, no corresponding Co peak is observed. It is presumed that Co is uniformly doped into MnO_x_, and the crystal form of MnO_x_ is not changed [33]. The corresponding FT-IR spectrum is illustrated in Figure S2, and the peak at 530 cm^−1^ belongs to the stretching vibration of Co–O [34]. The peak at 3412 cm^−1^ is assigned to the O–H stretching vibration of H_2_O [35].

X-ray photoelectron spectroscopy (XPS) is used to analyze the elemental content and chemical state of CoMnO_x_. The XPS survey spectrum (Figure S3A) shows the elements of Co, Mn, and O observed on CoMnO_x_ surface. As shown in Figure S3B, the peaks of 641.91 eV and 653.54 eV should be ascribed to Mn 2p_3/2_ and Mn 2p_1/2_ of Mn^3+^, and the peaks of 643.10 eV and 654.38 eV are attributed to Mn 2p_3/2_ and Mn 2p_1/2_ of Mn^2+^ [13,15]. Figure S3C shows that the peaks of 796.94 eV and 782.79 eV are associated with Co 2p_1/2_ and 2p_3/2_ of Co^2+^, and the peaks at 795.37 eV and 780.37 eV for Co 2p_1/2_ and 2p_3/2_ are associated with Co^3+^ [34,35]. The content of Co^3+^/(Co^2+^+Co^3+^) is as high as 71.09%, indicating that Co^3+^ is the main species of Co in the obtained CoMnO_x_. Thus, the redox ability of CoMnO_x_ is attributed to the different valence states of Mn and Co elements. The surface O species are displayed in Figure S3D. Three kinds of O species, namely surface lattice oxygen (O_l_), oxygen vacancy (O_v_), and chemisorbed oxygen (O_ads_), are presented. O_l_, O_v_, and O_ads_ are located at 529.85 eV, 531.29 eV, and 532.35 eV, respectively [36]. The O_l_ species accounts for 72.97% of the total surface oxygen, and O_v_ and O_ads_ account for 17.15% and 9.88%, respectively.

### 3.2. Enzyme-Like Catalytic Activities and Mechanisms

#### 3.2.1. Enzyme-Like Catalytic Activities

**Figure 2:**
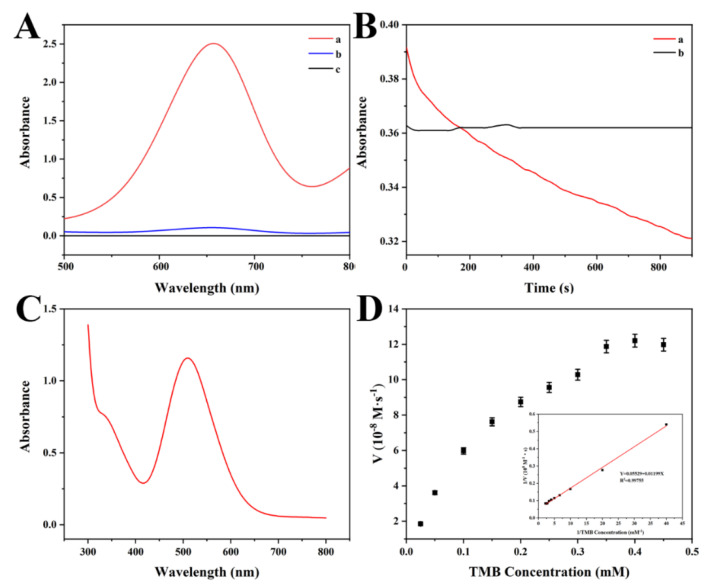
(**A**) UV-Vis absorbance spectra of different treatments for oxidase-like activity (a: CoMnO_x_ + TMB, b: TMB, c: CoMnO_x_) after incubation at 40 °C and pH 4.5; (**B**) UV-Vis of different treatments for catalase-like activity (a: CoMnO_x_ + H_2_O_2_, b: H_2_O_2_) after incubation at 40 °C and pH 4.5; (**C**) UV-Vis of the mixture of CoMnO_x_ suspension, 50 mM MES buffer (pH 7.0), 100 μL 2,4-DCP (1.0 mg·mL^−1^), and 4-AP for laccase-like activity after incubation for 90 min at 37 °C; (**D**) study on the steady-state kinetics of CoMnO_x_ after incubation at 40 °C for 20 min (Lineweaver–Burk reciprocal plot, *n* = 3).

The oxidase-like activity of CoMnO_x_ was evaluated (shown in Figure 2A). It shows that no absorption peaks at 652 nm are found with only TMB or CoMnO_x_ in the reaction system. However, a characteristic absorption peak is observed in the presence of CoMnO_x_ + TMB, indicating that TMB can be oxidized into oxTMB due to the oxidase-like activity of CoMnO_x_. In addition, the condition optimization experiments are displayed in Figure S4. As for the effect of pH on the oxidase-like activity of CoMnO_x_, the intensity of absorbance increases with pH from 2.5 to 4.5, and then it decreases after a pH above 4.5. Thus, the optimal pH is 4.5 (Figure S4A). The effect of temperature on the oxidase-like activity is the same as that of pH, and the highest intensity of absorbance is obtained at 40 °C (Figure S4B). As for the effect of CoMnO_x_ concentration, the intensity of absorbance increases from 0.01 mg·mL^−1^ to 0.05 mg·mL^−1^ and then remains unchanged. Thus, the optimized concentration of CoMnO_x_ is 0.05 mg·mL^−1^ (Figure S4C). For the effect of TMB concentration, the intensity of absorbance increases until a TMB concentration of up to 0.35 mM (Figure S4D). Therefore, the highest oxidase-like activity of CoMnO_x_ is presented at pH 4.5 and 40 °C. Meanwhile, the optimized concentrations of CoMnO_x_ and TMB are 0.05 mg·mL^−1^ and 0.35 mM, respectively.

The catalase-like activity of CoMnO_x_ was determined by the degradation of H_2_O_2_. The results show that the intensity of absorbance is not changed with the increase in time when only H_2_O_2_ exists. This indicates that the H_2_O_2_ is not degraded. However, the intensity of absorbance decreases as time increases for the CoMnO_x_ + H_2_O_2_ system, and bubbles are produced simultaneously, proving that CoMnO_x_ has catalase-like activity (Figure 2B).

As for the laccase-like activity of CoMnO_x_, 2,4-DCP was used as a substrate, and the color of the CoMnO_x_ + 2,4-DCP + 4-AP system changes from colorless to red. The intensity of absorbance presents an increase first and then a decreasing trend from 400 nm to 600 nm, and a strong UV-Vis absorption peak at 510 nm is observed in Figure 2C. This indicates that CoMnO_x_ has specific laccase-like activity, which may be applied in the field of biosensing [37].

#### 3.2.2. Kinetic Studies

The oxidase-like catalytic efficiency was investigated by steady-state kinetics under optimal conditions [21]. Typical Michaelis–Menten curves were studied under different concentrations of TMB [38]. The typical Lineweaver–Burk equation is Y = 0.05529 + 0.01199X. Based on the Lineweaver–Burk equation, the *K*_m_ of CoMnO_x_ is 0.0022 mM and the *V*_max_ value is 0.1809 µM·s^−1^ (Figure 2D). Compared to other literature (Table S1), the *K*_m_ value of CoMnO_x_ is lower than that of other nanozymes, indicating that CoMnO_x_ has stronger affinity toward TMB.

#### 3.2.3. Catalytic Mechanisms

**Figure 3:**
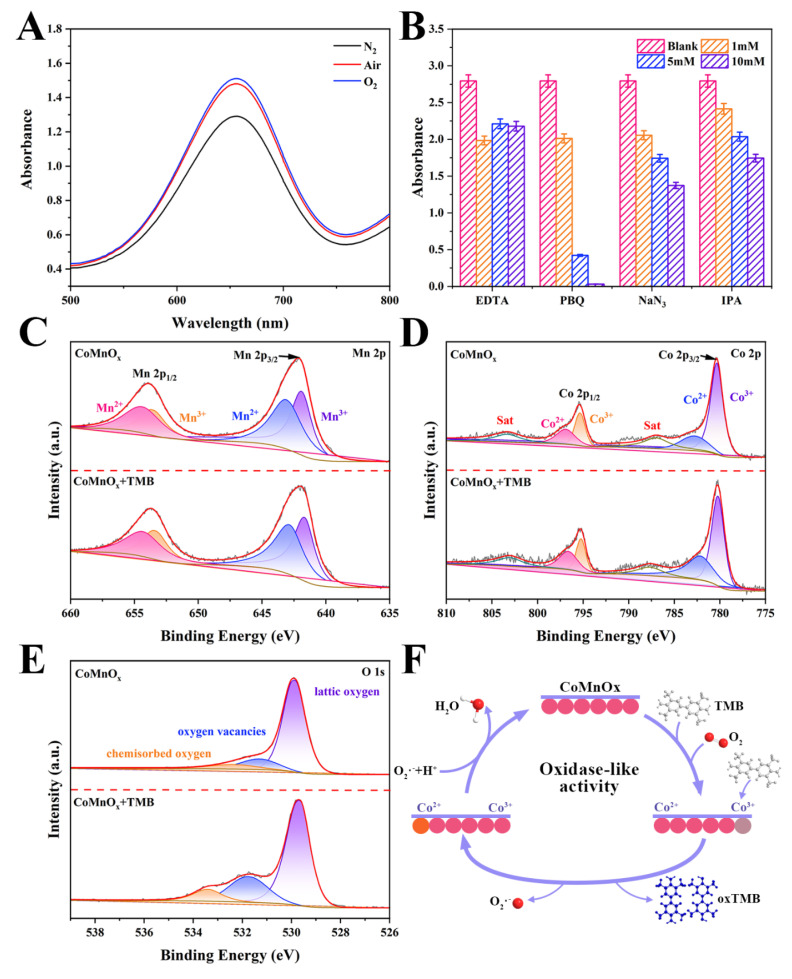
(**A**) UV-Vis spectra of CoMnO_x_ + TMB in N_2_-, air-, or O_2_-saturated systems after incubation at 40 °C and pH 4.5; (**B**) relative activity of different scavengers in the CoMnO_x_ + TMB system (*n* = 3); (**C**–**E**) XPS characterization: (**C**) Mn 2p, (**D**) Co 2p, (**E**) O 1s; (**F**) possible mechanism of CoMnO_x_ showing oxidase-like activity.

N_2_ purging experiments and reactive oxygen species (ROS) scavenging experiments were carried out to investigate the catalytic oxidation mechanism. The role of dissolved oxygen in the catalytic oxidation reaction of CoMnO_x_ was studied under different atmospheric conditions (O_2_, N_2_, and air). The absorbance increased under the O_2_ atmosphere (Figure 3A). However, the catalytic activity was inhibited under the N_2_ atmosphere. This indicates that O_2_ plays a key role in the oxidase process of CoMnO_x_.

The oxidase-like catalytic processes of CoMnO_x_ were further determined by changing different scavengers [39]. Different radical scavengers were employed to investigate reactive oxygen species (ROS) formed during the oxidase-like reaction. Ethylenediaminetetraacetic acid (EDTA), isopropanol (IPA), *p*-benzoquinone (PBQ), and sodium azide (NaN_3_) were used as scavengers of oxygen vacancy (OV), hydroxyl radical (·OH), superoxide anion (O_2_^−^), and singlet oxygen (^1^O_2_), respectively. The absorbances decreased when increasing the concentration of the scavengers (EDTA, IPA, PBQ, and NaN_3_), indicating that the catalytic oxidation of CoMnO_x_ is related to OV and the other three kinds of ROS (Figure 3B). Compared with the results of other radical scavengers, the absorbance was most severely decreased after PBQ addition. The intensity of absorbance almost dropped to zero when the PBQ was up to 10 mM. The results show that O_2_^−^ plays the most important role.

Figure 3C–E and Table S2 show that the Co^2+^/Co^3+^ ratio increases from 0.407 to 0.726 during the reaction and, thus, the content of Co^2+^ increases significantly in the CoMnO_x_ + TMB system. This is because CoMnO_x_ can catalyze TMB to oxTMB, making an electron transfer from TMB to Co^3+^. The surface Mn^2+^/Mn^3+^ ratio of CoMnO_x_ slightly increases during catalytic oxidation. According to Table S3, the proportion of oxygen vacancies increases from 17.15% to 26.57% during the catalytic reaction, while the proportion of surface lattice oxygen decreases from 72.97% to 64.17%. The increase of oxygen vacancy during the reaction might optimize the adsorption energy of the reaction substrate on the surface of CoMnO_x_. The decrease of surface lattice oxygen indicates that lattice oxygen can take part in the oxidase reaction. O_2_ molecules adsorbed on the surface of CoMnO_x_ are activated to produce a large amount of O_2_^−^. Then, O_2_^−^ can extract acidic hydrogen from TMB to produce the blue product oxTMB. Meanwhile, TMB is adsorbed and oxidized to the blue product oxTMB via Co^3+^, and Co^3+^ is reduced to Co^2+^ via electron transfer. Finally, the CoMnO_x_ nanozyme is regenerated (Figure 3F). Therefore, reasonable mechanisms for the oxidase-like activity are speculated and presented as follows:

Mn^2+^ + O_2_ → Mn^3+^ + O_2_

Co^3+^ + TMB → Co^2+^ + oxTMB

O_2_^−^ + TMB → oxTMB

Co^2+^ + Mn^3+^ → Co^3+^ + Mn^2+^

### 3.3. Colorimetric Sensing

**Figure 4:**
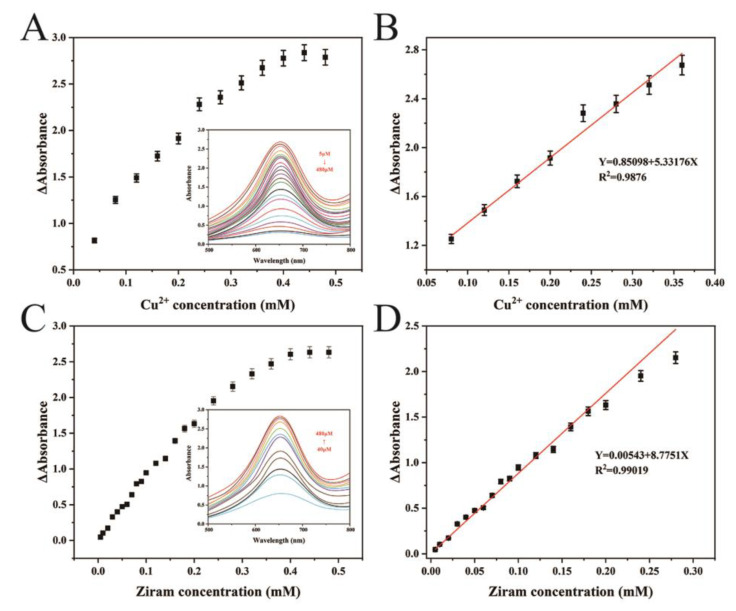
(**A**) Effects of various concentrations of ziram on the oxidase-like activity of the mixture of 210 μL TMB, 150 μL CoMnO_x_, and HAc-NaAc buffer at 652 nm (*n* = 3); (**B**) corresponding calibration curve of ziram; (**C**) effects of various concentrations of Cu^2+^ on the oxidase-like activity of the mixture of 480 μL ziram (4 mM), 210 μL TMB, 150 μL CoMnO_x_, and HAc-NaAc buffer at 652 nm (*n* = 3); (**D**) corresponding calibration curve of Cu^2+^.

Colorimetric methods for the analysis of ziram and Cu^2+^ were further established using the oxidase activity of CoMnO_x_. Compared with the absorbance of the TMB + CoMnO_x_ system, the absorbance at 652 nm decreases slowly with the increasing concentration of ziram in the range of 5~280 μM (Figure 4A). Figure 4B reveals that the linear relationship is Y = 0.0054 + 8.7715X (R^2^ = 0.9902). According to the LOD equation (3*δ*/*S*, where *δ* is the standard deviation of the blank solution and *S* is the slope of the calibration curve), the LOD of ziram is 1.475 μM. These results reveal that the sensing platform for ziram has a wider linear range compared to the previous reports listed in Table S4. Figure 4C shows that the absorbance of CoMnO_x_ + TMB + ziram + Cu^2+^ gradually increases with the increase of Cu^2+^ content. The UV absorbance of the reaction system can be restored by adding Cu^2+^, which indicates that the interaction of ziram and Cu^2+^ may exist. Figure 4D shows that the linear range is 80~360 μM and the linear relationship is Y = 0.8510 + 5.3318X (R^2^ = 0.9876). In comparison with the other studies shown in Table S5, the detection range of Cu^2+^ is relatively wide. 

### 3.4. Detection Mechanisms

**Figure 5:**
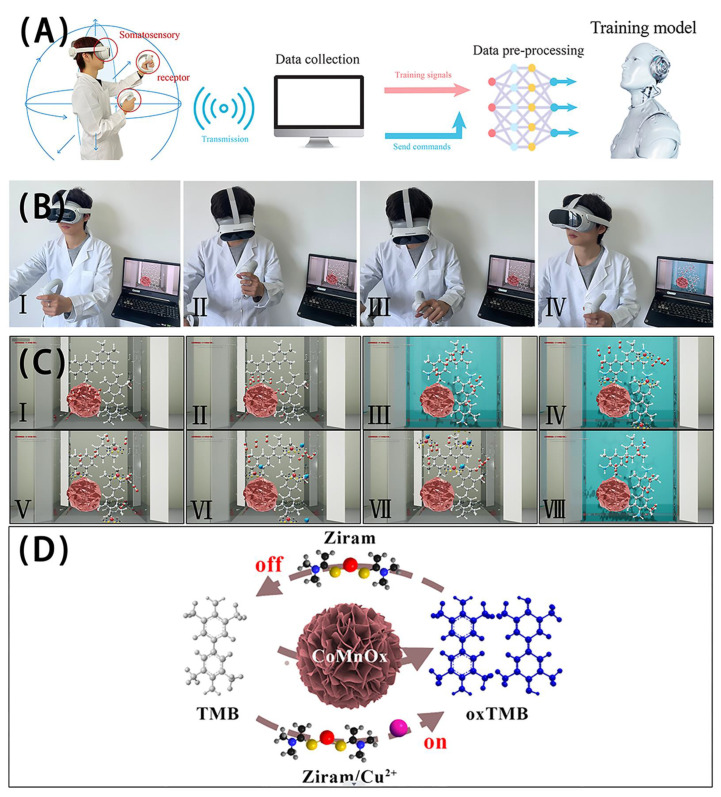
(**A**) Flow chart of virtual space establishment and design; (**B**) interactive experience of virtual reality design; (**C**) key training items in the virtual reality space. Step Ⅰ: O_2_ adsorption; Ⅱ: O_2_^−^ production; Ⅲ: TMB oxidation by O_2_^−^; Ⅳ and Ⅴ: interaction of ziram and oxTMB; Ⅵ: inhibitory effect of TMB oxidation by O_2_^−^ after adding ziram; Ⅶ: interaction of Cu^2+^ and ziram; Ⅷ: recovery of oxidase-like activity; (**D**) detection mechanisms of ziram and Cu^2+^ based on CoMnO_x_.

In order to explain the detection mechanism clearly, the detection processes of ziram and Cu^2+^ are revealed by virtual reality (VR) technology. A three-dimensional spatial model of the detailed reaction process is established using a computer system (Figure 5A). The user can wear VR glasses to observe and experience the specific detection process (Figure 5B). Figure 5C displays the detail detection mechanism. Firstly, O_2_ is adsorbed on the active sites of CoMnO_x_ (Ⅰ). Secondly, O_2_ is catalyzed by CoMnO_x_ to produce O_2_^−^ due to the electron transfer of Co and Mn elements (Ⅱ). Thirdly, TMB is oxidized by O_2_^−^ to form oxTMB. Meanwhile, the color is changed from colorless to blue (Ⅲ). Fourthly, the interaction between ziram and oxTMB is formed, and the color is returned to colorless after adding ziram (Ⅳ and Ⅴ). According to the structure of ziram (Figure S5), the negative charge center N atom of ziram can produce an interaction with the oxTMB cationic radicals. At the same time, the two strong electron-donating methyl groups of ziram and the electronegative center of N atom, can transfer the electron to oxTMB and make the blue color lighter. Moreover, the produced O_2_^−^ cannot oxidize the reduced oxTMB due to the interaction between ziram and oxTMB. Fifthly, the coordination effect of Cu^2+^ and ziram can get rid of the interaction of ziram and oxTMB, making the color return to the blue. Moreover, the detection mechanisms of ziram and Cu^2+^ based on CoMnO_x_ are also revealed in Figure 5D and the supporting video.

### 3.5. Smartphone Platform for Target Analysis

**Figure 6:**
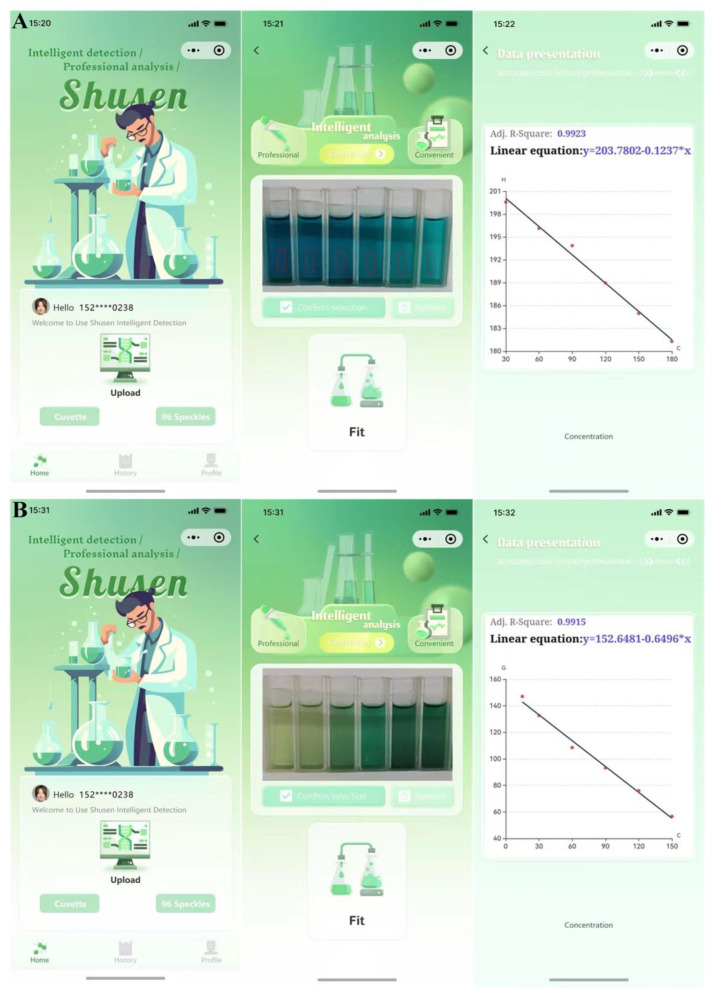
Intelligent detection platform for (**A**) ziram and (**B**) Cu^2+^.

The object detection model is established based on the object recognition and positioning algorithm of deep learning. The colors of the cuvette photos are automatically extracted and classified by the model, and the corresponding Red-Green-Blue (RGB) or Hue-Saturation-Value (HSV) values are calculated. Users only need to input the corresponding concentrations of detected objects and the number of samples, and the target linear curves of RGB or HSV values and the concentrations of detected objects are fitted automatically. As shown in Figure 6, the blue color becomes lighter and brighter when ziram is added into the system. The photos of the colorimetric results are uploaded to the smartphone, and the values of RGB and HSV can be recognized automatically by the artificial intelligence program. Compared with other fitting results, the H value and ziram concentration are used for linear fitting due to the highest correlation coefficient. Finally, the linear equation of Y = 203.7802 − 0.1237X (R^2^ = 0.9923) is generated automatically (Figure 6A), which can be used for on-site and timely ziram detection. Similarly, the smartphone can also detect the concentration of Cu^2+^. Compared with other fitting results, the G value and Cu^2+^ concentration present the highest correlation coefficient. The linear equation of Y = 152.6481 − 0.6496X (R^2^ = 0.9915) is obtained for Cu^2+^ detection (Figure 6B). 

### 3.6. Real Sample Analysis

Environmental samples of ziram and Cu^2+^ are simulated by the standard addition method to prove the practicability of the platform. Compared with the standard values of ziram, the recovery ranges from 96.60% to 102.18%, and the relative standard deviations (RSD) range from 1.04% to 3.67% (*n* = 3) (Table S6). As for Cu^2+^ detection (Table S7), the recovery is between 98.73% and 100.42%, and the RSD ranges from 0.52% to 4.69% (*n* = 3). These results confirm the applicability of the intelligent platform in real sample detection.

### 3.7. Selectivity and Stability Assay

Interfering pesticide substances of 2,4-dichlorophenoxyacetic acid, glufosinate ammonium, ethrel, carbendazim, acetamiprid, and atrazine are measured based on the detection platform (Figure S6A). In addition, the concentrations of these species are 100 times higher than that of ziram. The absorbance of each interfering substance is unchanged compared to the blank, except ziram. These results indicate that CoMnO_x_ has excellent specificity for ziram detection. To evaluate Cu^2+^ sensing selectivity, Na^+^, Mn^2+^, Mg^2+^, Zn^2+^, Ca^2+^, K^+^, Al^3+^, Pb^2+^, and Cd^2+^ (their concentrations are 100 times higher than that of Cu^2+^) are used as interfering substances (Figure S6B). In comparison with other metal ions, only the combination of Cu^2+^ and ziram can restore the absorption. These results show that the sensor has a high selectivity for Cu^2+^ detection.

The stability and reproducibility of the oxidase activity of CoMnO_x_ were also tested. As shown in Figure S5C, CoMnO_x_ can maintain high activity even after 60 days, indicating that CoMnO_x_ has good stability. In addition, 10 batches of CoMnO_x_ nanoflowers are synthesized repeatedly and their enzyme activities are measured (Figure S6D). The results show that the relative standard deviation (RSD) of different batches of CoMnO_x_ nanoflowers is only 3.19%, which indicates that CoMnO_x_ has good reproducibility (Figure S6D).

## 4. Discussion

The CoMnO_x_ nanozyme is designed and synthesized by hydrothermal reaction and high-temperature calcination methods. The obtained flower-shaped CoMnO_x_ presents three kinds of nanozyme activities, namely oxidase-, catalase-, and laccase-like activities. Among them, the oxidase-like activity is studied in detail. In addition, the highest oxidase-like activity of CoMnO_x_ is presented at a pH of 4.5 and temperature of 40 °C. Moreover, the oxidase-like catalytic efficiency was investigated by steady-state kinetics under optimal conditions. The *V*_max_ value is 0.1809 µM·s^−1^ and the *K*_m_ of CoMnO_x_ is 0.0022 mM, which is much lower than that of other nanozymes due to their stronger affinity toward TMB. The high oxidase-like activity is attributed to the changed valence state of Co and Mn elements. During the reaction, the Co^2+^/Co^3+^ ratio increases from 0.407 to 0.726 and the surface Mn^2+^/Mn^3+^ ratio of CoMnO_x_ is also slightly increased. Moreover, the proportion of oxygen vacancies increases from 17.15% to 26.57% during the catalytic reaction, while the proportion of surface lattice oxygen decreases from 72.97% to 64.17%. In detail, O_2_ molecules adsorbed on the surface of CoMnO_x_ are activated to produce a large amount of ROS (·OH, O_2_^−^, and 1 O_2_), especially O_2_^−^. According to the changes of oxygen species, some lattice oxygen can take part in the oxidase reaction to produce O_2_^−^, and lattice oxygen is changed to oxygen vacancies. O_2_^−^ can extract acidic hydrogen from TMB to produce the blue product oxTMB. Meanwhile, TMB is adsorbed and oxidized to the blue product oxTMB via Co^3+^, and Co^3+^ is reduced to Co^2+^ via electron transfer. CoMnO_x_, as an oxidase-like catalyst, can remain unchanged after the reaction. In other words, the nanozyme is regenerated after the reaction.

Based on its excellent oxidase activity, an “on-off-on” colorimetric sensor for the detection of ziram and Cu^2+^ has been developed. The absorbance at 652 nm decreases slowly after adding ziram. The reason might be that the active sites of CoMnO_x_ are covered by ziram. However, the absorbance of the CoMnO_x_ + TMB + ziram is restored gradually after the addition of Cu^2+^, indicating that the active sites of CoMnO_x_ are uncovered due to the interaction of ziram and Cu^2+^. The linear detection ranges for ziram and Cu^2+^ are 5~280 μM and 80~360 μM, and the detection limits are 1.475 μM and 3.906 μM, respectively. The detection of ziram shows wider detection ranges and lower detection limits than that in other studies (Table S4), and the detection of Cu^2+^ also shows a relatively wide detection range compared with other studies (Table S5). Moreover, an intelligent detection platform has further been established by combining the colorimetric signals with a portable smartphone. The developed ziram and Cu^2+^ portable smartphone platform can be used in environment analysis instantaneously.

## 5. Conclusions

In summary, flower-like CoMnO_x_ has been successfully synthesized by hydrothermal synthesis and high temperature calcination. The obtained CoMnO_x_ presents OXD-, CAT-, and laccase-like activities. The reaction kinetics and mechanisms have been studied deeply. The reaction kinetic results show that CoMnO_x_ has a strong affinity toward TMB with a low *K*_m_ (0.0022 mM). The reaction mechanisms show that TMB can be oxidized to oxTMB by a large amount of generated O_2_^−^. Meanwhile, TMB is also oxidized directly to the blue product oxTMB via the high redox ability of Co species. Based on its excellent oxidase activity, an “on-off-on” colorimetric sensor for the detection of ziram and Cu^2+^ has been developed. The linear detection ranges for ziram and Cu^2+^ are 5~280 μM and 80~360 μM, and the detection limits are 1.475 μM and 3.906 μM, respectively. The detection of ziram and Cu^2+^ shows wider detection ranges and lower limits than that in other studies. Moreover, a ziram and Cu^2+^ portable smartphone platform has been constructed successfully and used in on-site and timely environment analysis.

## Data Availability

The date presented in this study are available in Supplementary Materials.

## Supplementary Materials

The following supporting information can be downloaded at: https://www.mdpi.com/article/10.3390/bios14040178/s1, Figure S1: XRD image of CoMnO_x_; Figure S2: FTIR spectrum of CoMnO_x_; Figure S3: (A–D) XPS full, (B) Mn 2p, (C) Co 2p, and (D) O 1s spectra of CoMnO_x_; Figure S4: Optimization of reaction conditions based on CoMnO_x_ oxidase-like activity: (A) pH; (B) temperature; (C) material concentration; (D) TMB concentration (*n* = 3); Figure S5: Potential interference of other substances for the detection of (A) ziram and (B) Cu^2+^; (C and D) stability and reproducibility of the nanozyme for target sensing; Table S1: Comparison of steady-state kinetic parameters for the oxidase-like activity of CoMnO_x_ and other nanozymes; Table S2: XPS analysis results of Mn 2p and Co 2p; Table S3: XPS analysis results of O 1s; Table S4: Comparison of different methods for the detection of ziram; Table S5: Comparison of different methods for the detection of Cu^2+^; Table S6: Assay results of ziram in soil and water samples; Table S7: Assay results of Cu^2+^ in soil and water samples. [40,41,42,43,44,45,46,47,48,49,50,51,52,53,54,55,56,57,58,59,60,61,62,63,64,65,66].
