# Supplementary material for: CoMnOx Nanoflower-Based Smartphone Sensing Platform and Virtual Reality Display for Colorimetric Detection of Ziram and Cu2+
Source: Biosensors (Basel). 2024 Apr 6;14(4):178. doi: 10.3390/bios14040178 (PMC11048373; doi:10.3390/bios14040178)
Supplement: Supplementary file 1 [file biosensors-14-00178-s001.zip › biosensors-2884809-supplementary.pdf]

$CoMnO_x$ Nanoflower-Based Smartphone Sensing Platform and Virtual Reality Display for Colorimetric Detection of Ziram and Cu^{2+}

Chang Song [1,†], Fangfang Wang [2,†], Xin Zhang [1], Yuanxia Ma [1], Yangyu Wu [3], Mingxia He [2], Xiangheng Niu [3,*] and Mengmeng Sun [2,*]

[1] School of Arts and Media, Sichuan Agricultural University, Chengdu 611130, China
[2] College of Science, Sichuan Agricultural University, Ya'an 625014, China
[3] School of Public Health, Hengyang Medical School, University of South China, Hengyang 421001, China
* Correspondence: niuxiangheng@usc.edu.cn (X.N.); 14391@sicau.edu.cn (M.S.)
† These authors contributed equally to this work.

Reagents and Chemicals

Cobaltous nitrate ($Co(NO_3)_2$), hydrogen peroxide (H_2O_2, 30%), tert-butanol (TBA), sodium azide (NaN_3), potassium permanganate ($KMnO_4$), dimethyl sulfoxide (DMSO), ethylenediamine tetraacetic acid disodium salt (EDTA), riboflavin, *p*-benzoquinone (PBQ), and ethanol (CH_3CH_2OH) were supplied by KeLong (Chengdu, China, http://www.cdkelong.com/). Ziram was purchased from Macklin Biochemical Co. Ltd. (Shanghai, China, http://www.macklin.cn/). 3,3',5,5'-Tetramethylbenzidine (TMB) was obtained from Shanghai Ryon Biological Technology Co. Ltd. (Shanghai, China, http://ruien.company.lookchem.cn/). All the chemicals used in experiments were analytically pure. All experiments used deionized water (18.25 $M\Omega\cdot cm^{-1}$).

Characterization Equipment

The crystal structure, morphological structure, elemental composition and surface functional groups were analyzed by X-ray diffraction (XRD, DX-2700, Dandong, China), transmission electron microscope (TEM, JSM4800F, JEOL, Japan), scanning electron microscope (SEM, JEOL-2100F, Japan), X-ray photoelectron spectroscope

(XPS, ESCALAB-250Xi, China), and Fourier transform infrared spectrometer (FTIR, Bruker Vertex 70, China), respectively.

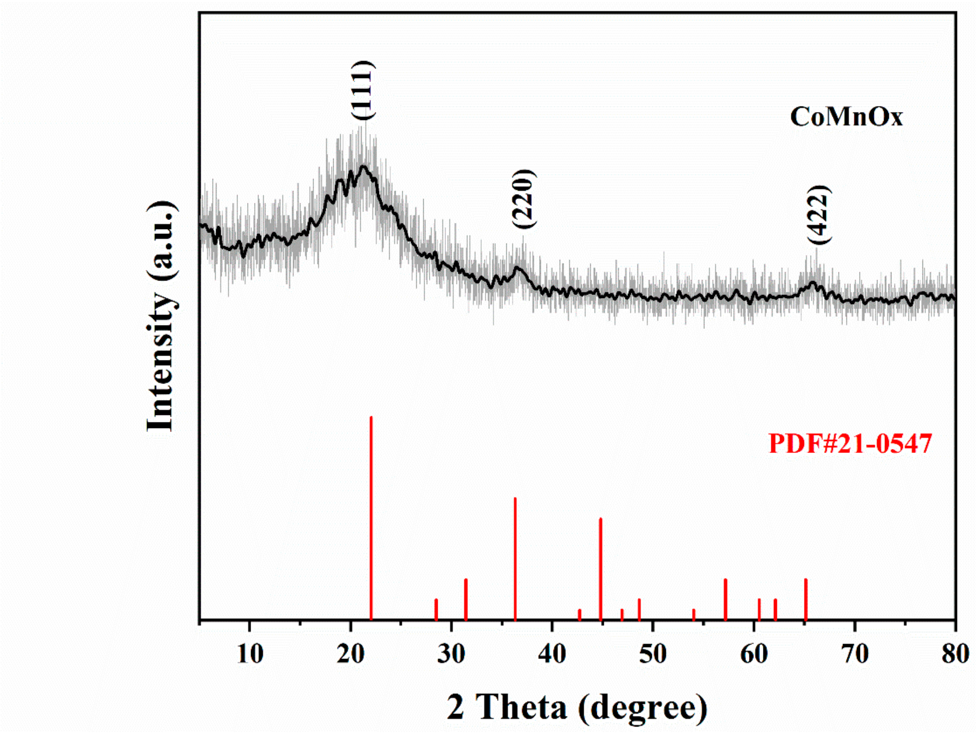

Figure S1. XRD image of $CoMnO_x$.

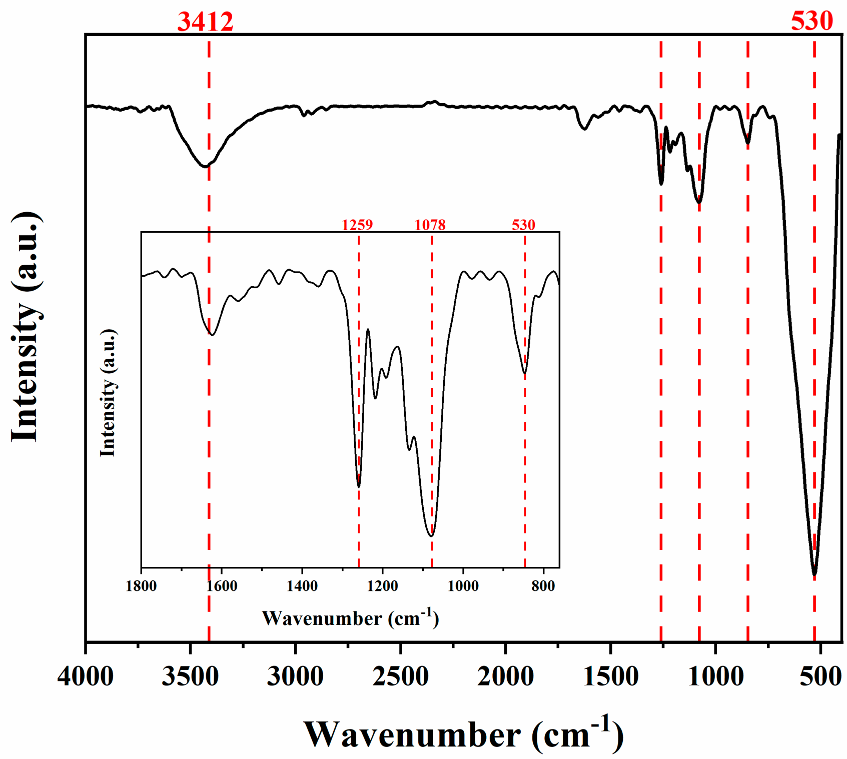

Figure S2. FTIR spectrum of $CoMnO_x$.

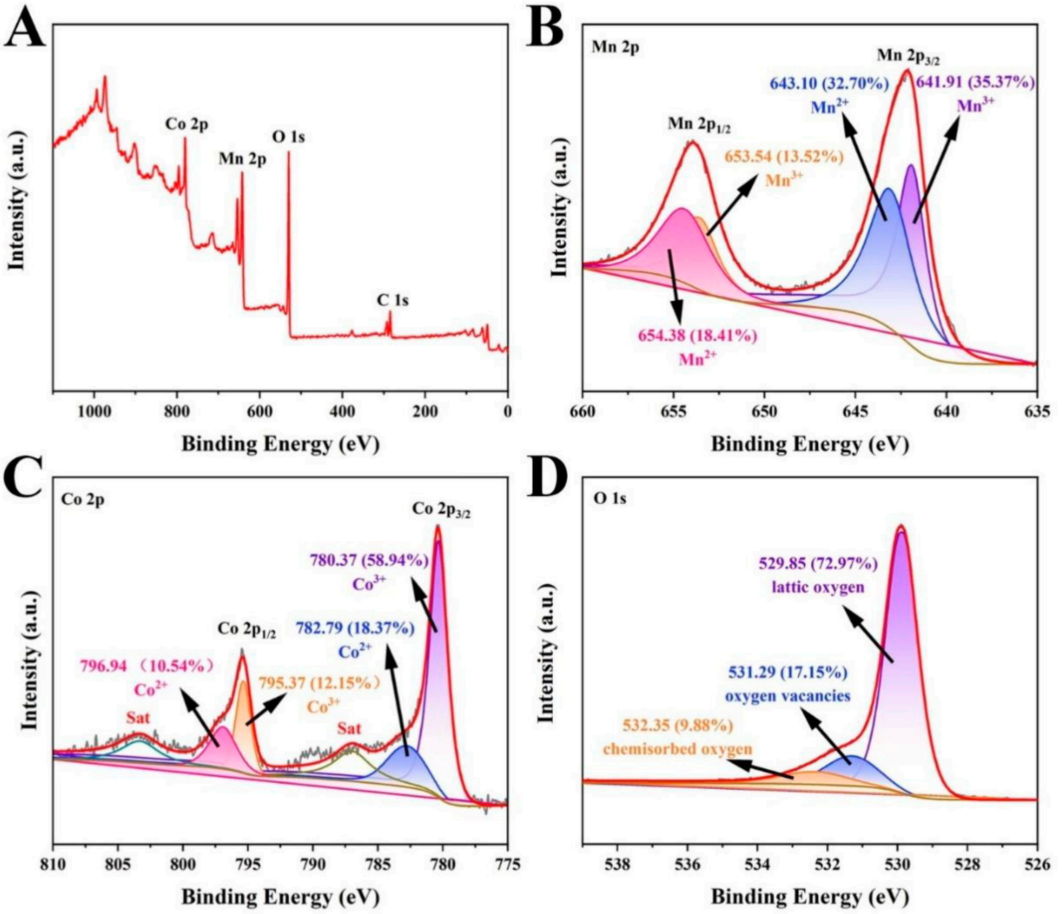

Figure S3. (A) XPS full spectrum; (B) Mn 2p, (C) Co 2p and (D) O 1s spectra of $CoMnO_x$.

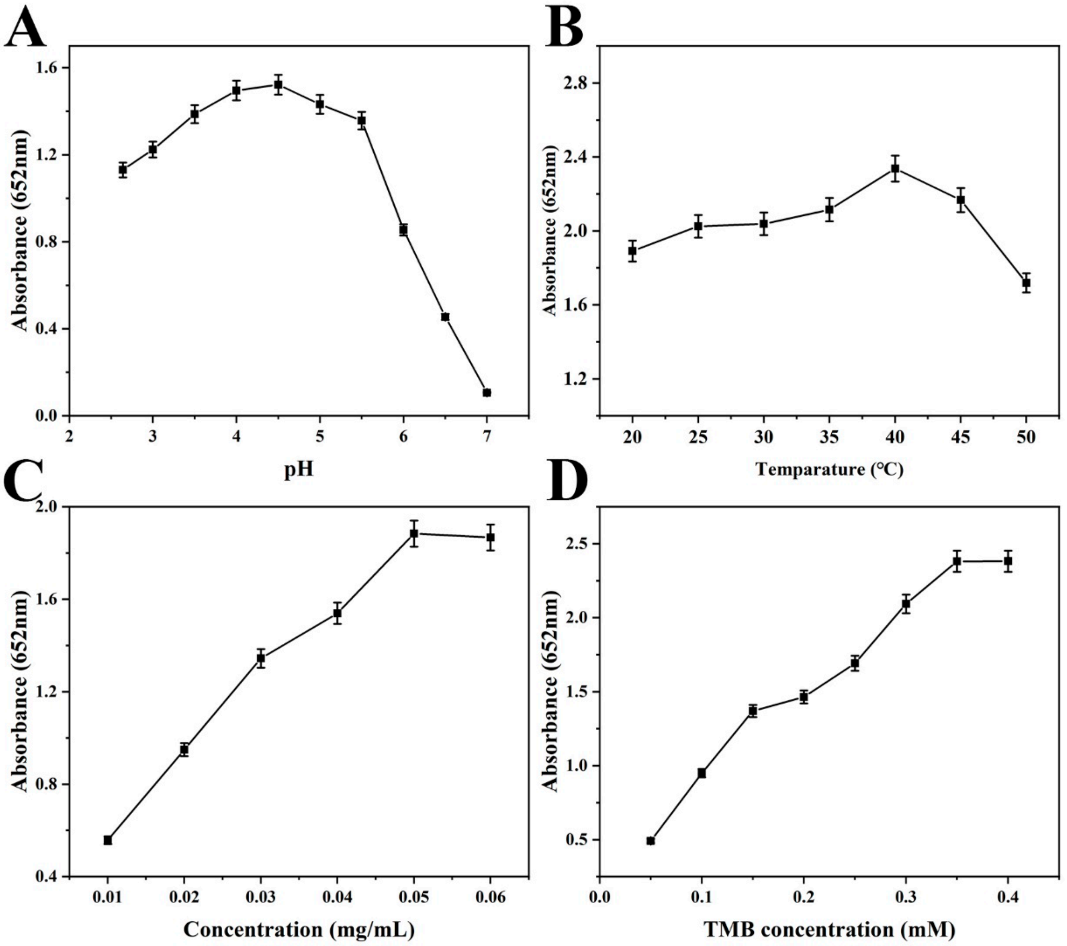

Figure S4. Optimization of reaction conditions for $CoMnO_x$ oxidase-like activity. (A) pH; (B) Temperature; (C) Material concentration; (D) TMB concentration (*n*=3).

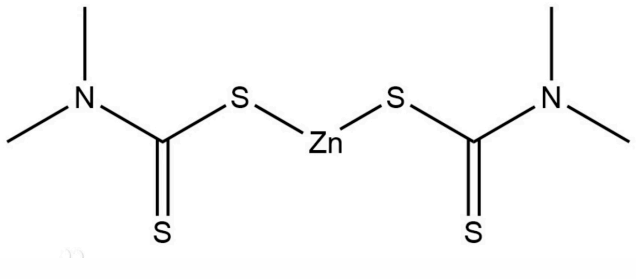

Figure S5. Chemical structure of Ziram

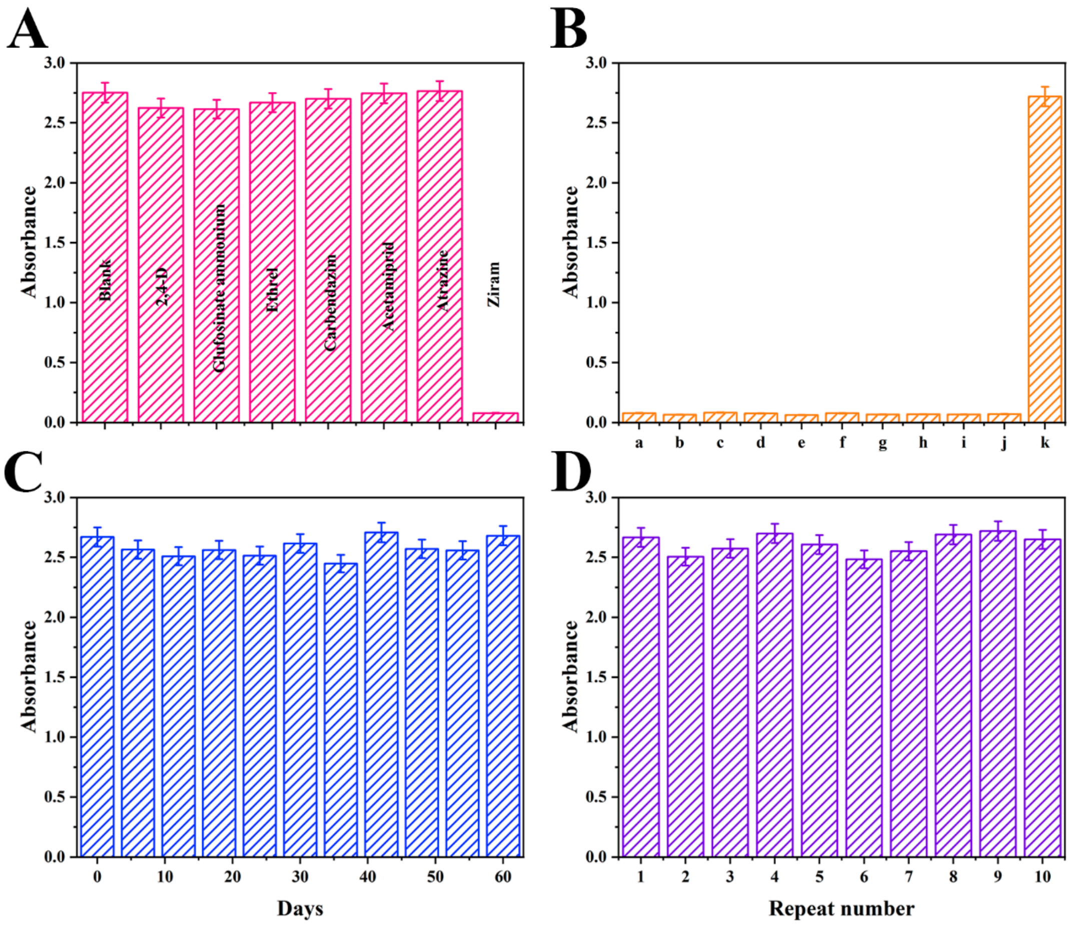

Figure S6. Potential interferences of other substances for the detection of (A) Ziram and (B) Cu^{2+} (a: control, b: Na^{+}, c: Mn^{2+}, d: Mg^{2+}, e: Zn^{2+}, f: Ca^{2+}, g: K^{+}, h: Al^{3+}, i: Pb^{2+}, j: Cd^{2+}, h: Cu^{2+}); (C) Sensor stability; (D) Reproducibility of our method (n=3).

Table S1. Comparison of steady-state kinetic parameters for the oxidase-like activity of $CoMnO_x$ and other nanozymes.

Nanozyme	K_m (mM)	V_{max} (μM·s^{-1})	Reference
Co_3O_4	0.0469	0.0459	**Error! Reference source not found.**
Co_3O_4 NPs	0.051	0.033	**Error! Reference source not found.**
Co-Fe LDH	0.218	0.263	**Error! Reference source not found.**
Co_3O_4/rGO	0.19	0.1071	**Error! Reference source not found.**
MnO_2 nanosticks	0.147	1.34	**Error! Reference source not found.**
FO@ZMFO@FM-MOG	0.291	0.149	**Error! Reference source not found.**
MOF(Co/2Fe)	0.199	0.39	**Error! Reference source not found.**
Fe_3O_4@MnO_2	0.12	–	**Error! Reference source not found.**
FeCo NPs@PNC	0.091	0.0939	**Error! Reference source not found.**
CNF/$MnCo_2O_{4.5}$	0.04	0.0645	**Error! Reference source not found.**

$CoMnO_x$	0.0022	0.1809	This work

Table S2. XPS analysis results of Mn 2p and Co 2p.

Sample	Mn^{2+}/Mn^{3+}	Co^{2+}/Co^{3+}
$CoMnO_x$	1.054	0.407
$CoMnO_x$+TMB	1.001	0.726

Table S3. XPS analysis results of O 1s.

Sample	Chemisorbed oxygen	Oxygen vacancy	Lattice oxygen
$CoMnO_x$	9.88%	17.15%	72.97%
$CoMnO_x$+TMB	9.26%	26.57%	64.17%

Table S4. Comparison of different methods for the detection of Ziram.

Material	Detection method	Linear range (μM)	LOD (μM)	Reference
p-AgSAE	Voltammetry	0.5-5	0.24	**Error! Reference source not found.**
Carbon dots	Fluorometry	0-16	1.80	**Error! Reference source not found.**
$CsPbBr_3$	Fluorometry	0.32-163.49	0.28	**Error!**

				**Reference source not found.**
Oct/Au@AgNPs	SERS[a]	0.33-32.70	0.050	**Error! Reference source not found.**
Silver nanoparticles	Colorimetry	0.64-2.4	0.49	**Error! Reference source not found.**
Pt/Co_3O_4	Colorimetry	5-50	3.36	**Error! Reference source not found.**
Copper foam	SEIRAS[b]	60-1000	58.87	**Error! Reference source not found.**
AuNPs	Fluorometry	0.052-0.50	0.052	**Error! Reference source not found.**
$CoMnO_x$	Colorimetry	5-280	1.475	This work

a: surface enhanced Raman spectroscopy

b: surface enhanced infrared absorption spectroscopy

Table S5. Comparison of different methods for the detection of Cu^{2+}.

Material	Detection method	Linear range (μM)	LOD (μM)	Reference
Chol-RN	Fluorometry	5-40	1.12	**Error! Reference source not found.**
Rhodamine-based derivative	Fluorometry	10-300	3.42	**Error! Reference source not found.**
Furfuraldehyde fluorescein hydrazone	Colorimetry	6.6-330	6.6	**Error! Reference source not**

				**found.**
Rhodamine B hydrazone	Fluorometry	2.63-200	2.63	**Error! Reference source not found.**
$Cu_2(BDC)_2(BPY)$	Fluorometry	50-250	0.005	**Error! Reference source not found.**
Ferrocene benzobisimidazole	Fluorometry	0-10	1.6	**Error! Reference source not found.**
Rhodamine-based fluorescent probe	Fluorometry	2-20	0.1	**Error! Reference source not found.**
Flu@Mea-CdS	Fluorometry	4-14	0.17	**Error! Reference source not found.**
Cu^{2+}-triethylamine	Colorimetry	0.0625-8.0	0.0625	**Error! Reference source not found.**
$CoMnO_x$	Colorimetry	80-360	3.906	This work

Table S6. Assay results of Ziram in soil and water samples (*n*=3).

**Sample**	**Add (μM)**	**Determined (μM)**	**RSD (%)**	**Recovery (%)**
Soil	0	0.87	1.04	–
	60	59.28	2.23	98.81
	90	91.03	2.70	101.14
	120	122.61	3.46	102.18

Water	0	0.87	1.04	–
	50	48.72	3.67	97.44
	100	100.84	1.60	100.84
	150	144.92	2.85	96.60

Table S7. Assay results of Cu^{2+} in soil and water samples (*n*=3).

**Sample**	**Add (μM)**	**Determined (μM)**	**RSD (%)**	**Recovery (%)**
Soil	0	0.94	1.21	–
	60	59.61	0.57	99.35
	90	89.33	2.16	99.25
	120	118.48	4.69	98.73
Water	0	0.94	1.21	–
	50	49.95	0.52	99.90
	100	100.42	1.42	100.42
	150	150.26	2.11	100.18
